# NA0D – The new Traumatic Dental Injury classification of the World Health Organization

**DOI:** 10.1111/edt.12753

**Published:** 2022-04-28

**Authors:** Stefano Petti, Jens Ove Andreasen, Ulf Glendor, Lars Andersson

**Affiliations:** ^1^ Department of Public Health and Infectious Diseases Sapienza University Rome Italy; ^2^ Department of Oral and Maxillofacial Surgery Copenhagen University Hospital Copenhagen Denmark; ^3^ Faculty of Odontology Malmö University Malmö Sweden

**Keywords:** global burden of disease, ICD‐11, international classification of diseases, traumatic dental injury, World Health Organization

## Abstract

An accurate, clear, and easy‐to‐use traumatic dental injury (TDI) classification and definition system is a prerequisite for proper diagnosis, study, and treatment. However, more than 50 classifications have been used in the past. The ideal solution would be that TDIs are adequately classified within the International Classification of Diseases (ICD), endorsed by the World Health Organization (WHO). TDI classification provided by the 11th Revision of the ICD (ICD‐11), released in 2018, and previous Revisions, failed to classify TDIs satisfactorily. Therefore, in December 2018, a proposal was submitted by Dr's Stefano Petti, Jens Ove Andreasen, Ulf Glendor, and Lars Andersson, to the ICD‐11, asking for a change of the existing TDI classification. Proposal #2130 highlighted the TDI paradox, the fifth most frequent disease/condition neglected by most public health agencies in the world, and the limits of ICD‐11 classification. Namely, injuries of teeth and periodontal tissues were located in two separate blocks that did not mention dental/periodontal tissues; infraction, concussion, and subluxation were not coded; most TDIs lacked description; and tooth fractures were described through bone fracture descriptions (e.g., comminuted, compression, and fissured fractures). These limitations led to TDI mis‐reporting, under‐reporting, and non‐specific reporting by untrained non‐dental healthcare providers. In addition, no scientific articles on TDIs, present in PubMed, Scopus, and Web‐of‐Science, used the ICD classification. Proposal #2130 suggested to adopt the Andreasen classification, the most widely acknowledged classification used in dental traumatology. The Proposal was reviewed by two WHO teams, two scientific Committees, one WHO Collaborating Center, and the Department of Non‐Communicable Disease Prevention at WHO headquarters, and it underwent two voting sessions. In March 2022, the Andreasen classification was accepted integrally. A new entity was generated, called NA0D, “Injury of teeth or supporting structures” (https://icd.who.int/browse11/l‐m/en#/http%3a%2f%2fid.who.int%2ficd%2fentity%2f1413338122). Hopefully, this will contribute to increasing the public awareness, and the dental profession's management, of TDIs.

## 1 THE WHO INTERNATIONAL CLASSIFICATION OF DISEASES AND TRAUMATIC DENTAL INJURIES

In 1891, the International Statistical Institute designed the so‐called Classification of Causes of Death, presented two years later, that received general approval and was adopted by several countries worldwide. The classification was updated periodically, and the 6th revision, called the International Classification of Diseases, Injuries, and Causes of Death, was endorsed by the World Health Organization (WHO) at the first World Health Assembly in 1948.^1^ The aim of the International Classification of Diseases (ICD) system was to provide a universally acknowledged tool for epidemiologic, health management, and clinical purposes.

To be able to diagnose, study, and treat injuries, a prerequisite is to use an accurate definition of the injury. Traumatic dental injuries (TDIs) have over the years been diagnosed and reported according to a variety of factors such as anatomy, etiology, pathology, therapeutic consideration, and degree of severity. The variety of diagnostic methods used has complicated accurate documentation of TDIs and interfered with the development of systematic comparisons between different studies and meta‐analyses of larger materials have been hindered.^2^ Such uncertainty could be responsible for the low awareness among non‐dental healthcare workers worldwide, which is a serious problem because TDI mis‐management could lead to serious sequelae, medico‐legal problems, and reduced quality of life for the patients.^3^

More than 50 TDI classification systems have been used in the past in the literature.^4^ The most commonly used classification was developed by Dr Jens Ove Andreasen in 1970^5^ with further modifications. This classification served as the basis for the TDI codification of the “Application of the International Classification of Diseases in Dentistry and Stomatology” (ICD‐DA), released by the WHO in 1995,^6^ an oral health related branch of the 10th Revision of the ICD (ICD‐10), endorsed at the 43rd World Health Assembly in 1990, and used by member states since 1994. However, the WHO classification was in many ways incomplete, and Dr Andreasen suggested certain entities to be added that were not included in the WHO system. The Andreasen classification has over the years become a global standard for clinicians and researchers, because of its appropriateness to be used in modern clinical practice and for comparing results from different studies. The strength of the Andreasen system is that it is very closely related to the specific tissue injuries, treatment methods, and later complications of dental injuries. Despite becoming the global standard for the clinic and research worldwide, the Andreasen classification was not completely taken into consideration for TDI classification in further ICD‐10 revisions.

In 2018, 28 years after the launching of the ICD‐10, the WHO released the 11th Revision of the ICD (ICD‐11) to allow the member states to start planning. ICD‐11 is a more modern digitized classification system that contains around 17,000 unique codes, more than 120,000 codable terms in a smart coding tool and it is entirely digital. By March 2022, 35 countries had already adopted ICD‐11. The ICD provides a common language that allows health professionals to share standardized information across the world. For this reason, it was important to correct the ICD‐11 according to the Andreasen classification system. Therefore, Dr's Stefano Petti, Jens Ove Andreasen, Ulf Glendor, and Lars Andersson decided to submit a proposal to modify the existing TDI classification that was present in ICD‐11.

The urge to re‐classify TDIs within the ICD system, based on solid public health, healthcare policy, and scientific motivations, was acknowledged internationally thanks to a Lancet Global Health publication in 2018,^7^ and was endorsed by the Editor‐in‐Chief of *Dental Traumatology*, Dr Paul Abbott.^8^ Nevertheless, the attempt to contact the ICD‐11 Revision Steering Group, the consultative expert authority in charge during the ICD revision process, was unsuccessful, because the Group ceased to exist in October 2016. Thus, the goal to modify the TDI related part of the ICD‐11 before its release, that occurred in June 2018, failed. The remaining alternative was to try with the ICD‐11 Maintenance Platform, a tool dedicated to post‐publication contributions (proposals, comments, and translations) to the classifications. Its consultation (available at: https://icd.who.int/dev11/Account/Login?ReturnUrl=%2Fdev11%2Fproposals%2Ff%2Ficd%2Fen%2FProposalList) is free to all, but requires registration. The evaluation of the proposals submitted to the Platform is complex and involves several WHO teams, including the Medical Scientific Advisory Committee, that reviews the proposals and is consulted on medical and scientific questions, and the Classification and Statistics Advisory Committee, that considers the proposals and makes the final recommendations on whether to accept or reject them. Several thousand proposals have been submitted so far, but only a few have been implemented and led to substantial ICD‐11 modifications. Indeed, the reviewing process of the proposal can be very complex and frustrating before everything is in order, and is open all the time to clinicians and researchers worldwide who can provide their comments, and are free to agree or disagree with the proposal by adding a green or a red flag. At the end of the process, the proposals are subjected to voting. Unanimous consensus must be achieved for proposal approval.

## 2 PROPOSAL #2130

The authors of the proposal opted for a type called “Complex Hierarchical Changes Proposal,” which was preferred over simpler types because of the many limits of the ICD‐DA/ICD‐10/ICD‐11 regarding TDI classification. The proposal was originated on December 3, 2018, and was numbered #2130; the full text is available in Appendix S1. These are the main issues.

1. *The paradox of TDIs*. TDI are one of the most frequent and neglected diseases at the same time. Indeed, TDIs affect more than one billion people worldwide, are the fifth most frequent disease/injury,^9^ and their treatment is extremely costly.^10^ Nevertheless, TDIs are ignored by the main public health organizations, such as the WHO and the US Centers for Disease Control and Prevention, and they are excluded from the list of the 300 most frequent diseases by the Global Burden of Disease Study. Such a situation also produces widespread unawareness toward TDIs.^7^, ^8^
2. *The limits of ICD‐11 (and ICD‐DA/ICD‐10) regarding TDIs*. The May 2021 release of ICD‐11 (available at: https://icd.who.int/browse/11/2021‐05/mms/en#452386362) shows the situation before the implementation of Proposal #2130. The following are just a few examples of TDI classification inadequacy. Traumatic injuries to teeth were located in a block called “Fracture of skull or facial bones,” with no mention of fractures of dental tissues. Traumatic injuries to periodontal tissues, imprecisely called “Dislocation of tooth,” were located in another block called “Dislocation or strain or sprain of joints or ligaments of head.” Astonishingly, periodontal tissues were ignored and were assimilated to joints and ligaments. Thus, at first sight, untrained healthcare providers could mistakenly think that TDIs were excluded from the ICD‐11 system, thus hampering or even preventing reporting. Some TDIs, namely infraction, concussion, and subluxation, were not coded at all, and, therefore, could not be reported or were reported using non‐specific codes. TDI entities were not defined, thus making it impossible their accurate coding by untrained healthcare providers, who could use non‐specific codes, such as “Unspecified injury of head,” “Injury, unspecified,” “Open wound of head, part unspecified.” Macroscopic mistakes were also present ‐ such as the description of tooth fracture sub‐types, that used the descriptions of bone fracture sub‐types, namely buckle, burst, comminuted, compression, dislocated, and fissured fractures. Definitively, these descriptions did not apply to teeth.
3. *Limited TDI coding associated with inaccurate reporting*. Actually, this is a chicken‐egg dilemma. Was it the inaccurate TDI classification by the WHO that produced unawareness toward TDIs, or was it the general unawareness toward TDIs that produced inaccurate TDI classification by the WHO? Solving this enigma is probably impossible. However, the two phenomena were associated. One example is the situation in general (i.e., non‐dental) Emergency services, where TDI treatment delay occurs almost systematically whenever dental healthcare providers are unavailable,^11^ because just a handful of physicians working in Emergency services can assess and treat TDIs appropriately,^12^ and the awareness of non‐dental Emergency healthcare providers toward TDIs is unsatisfactory.^3^ Indeed, only one fourth of TDIs were correctly classified in Emergency services in a UK District General Hospital, where the ICD system was used, while one fifth received the least specific codes (“Unspecified injury of head,” “Injury, unspecified”), with no information as to the cause, the nature, and the needed treatment. The situation regarding traumatic injuries to periodontal tissues was similar, with codes, such as “Open wound of other part of head,” “Open wound of unspecified body region,” and “Open wound of head, part unspecified” that were prevailing over specific codes. Finally, another one fourth of TDIs were not coded at all and received system‐generated codes.^13^
  Inadequacy of the ICD‐11 (and ICD‐DA/ICD‐10) in classifying TDIs is also evident from the scientific standpoint, as there were no TDI‐related scientific articles, present in PubMed, Web‐of‐Science, and Scopus, that used the ICD system to classify TDIs, up to 2018.
4. *The core of Proposal #2130*. The authors proposed to cancel all the codes related to TDIs and generate a new independent block, specific for teeth and periodontal tissues. This change was considered complex, but assumed to allow untrained healthcare providers to diagnose TDIs properly, to provide better treatments, and to uniform scientific studies on TDIs. The proposed name of the block was “Traumatic Dental Injuries” and the proposed classification was the Andreasen classification, as used in most healthcare and scientific settings worldwide for more than 50 years (Table 1).

**TABLE 1:** Traumatic dental injury classifications. Proposal #2130 submitted to the Maintenance Platform of the International Classification of Diseases 11th Revision (ICD‐11) in 2018, and version implemented by the World Health Organization at the end of the reviewing process in 2022

	Proposal #2130	Implemented version	ICD‐11 Code
Block name	Traumatic Dental Injuries	Injury of teeth or supporting structures	NA0D
First group	Injuries to the hard dental tissues and the pulp	Injury of hard dental tissues and pulp	NA0D.0
	Enamel infraction^a^	Enamel infraction	NA0D.00
	Enamel fracture	Enamel fracture	NA0D.01
	Enamel‐dentin fracture	Enamel‐dentin fracture	NA0D.02
	Complicated crown fracture	Complicated crown fracture	NA0D.03
	Uncomplicated crown‐root fracture	Uncomplicated crown‐root fracture	NA0D.04
	Complicated crown‐root fracture	Complicated crown‐root fracture	NA0D.05
	Root fracture	Root fracture	NA0D.06
		Other specified injury of hard dental tissues and pulp	NA0D.0Y
		Injury of hard dental tissues and pulp, unspecified	NA0D.0Z
Second group	Injuries to the periodontal tissues	Injury of periodontal tissues	NA0D.1
	Concussion^a^	Concussion of periodontal tissue	NA0D.10
	Subluxation^a^	Subluxation of tooth	NA0D.11
	Extrusive luxation	Extrusive luxation of tooth	NA0D.12
	Lateral luxation	Lateral luxation of tooth	NA0D.13
	Intrusive luxation	Intrusive luxation of tooth	NA0D.14
	Avulsion	Avulsion of tooth	NA0D.15
		Other specified injury of periodontal tissues	NA0D.1Y
		Injury of periodontal tissues, unspecified	NA0D.1Z
Other groups		Other specified injury of teeth or supporting structures	NA0D.Y
		Injury of teeth or supporting structures, unspecified	NA0D.Z

^a^ Entities that were not classifiable using the ICD system before the revision.

*History of the Proposal #2130*. The Proposal was discussed by the four authors during the year 2018 and entered in the Maintenance Platform on December 3, 2018. The reviewing process was long, but was deemed necessary, because of the complexity of the proposed changes, and because there were many thousands of submitted proposals to evaluate, with just a handful of them being approved. This process involved the Teams 3 and 6 of WHO, the WHO Collaborating Center for Quality Improvement and Evidence‐based Dentistry (QED WHO CC) at the New York University, the Department of Prevention of Noncommunicable Diseases (NCDs) at WHO Headquarter in Geneva, the Medical Scientific Advisory Committee (MSAC), and the Classification and Statistics Advisory Committee (CSAC), with two voting sessions (Table 2). Excerpts of the main comments to Proposal #2130 are in Appendix S2.

**TABLE 2:** History of Proposal #2130

Date	Event
3 December 2018	Registration of Proposal #2130 as “Complex Hierarchical Changes” to the Maintenance Platform of the ICD‐11
6 January 2019	Proposal submitted to the Maintenance Platform
6 March 2019	Proposal review by the Medical and Scientific Advisory Committee of the WHO Family of International Classifications (MSAC)
6 February 2020	Proposal sent back to the WHO Family of International Classifications by the MSAC
2 March 2021	Proposal review by the Classification and Statistics Advisory Committee of the WHO Family of International Classifications (CSAC)
3 March 2021	Opinion requested to the WHO Collaborating Center for Quality Improvement and Evidence‐based Dentistry (QED WHO CC), New York University, US^a^
4 March 2021	Comments provided by Benoit Varenne, Dental Officer at the Prevention of Noncommunicable Diseases (NCDs) Department, WHO, HQ, Geneva^a^
5 May 2021	Comments provided by the CSAC Small Group^a^
5 May 2021	CSAC voting the Proposal (first round)
4 July 2021	CSAC voting Summary Yes = 5 No = 0 Undecided = 5
27 July 2021	CSAC voting the Proposal (second round)
9 August 2021	Letter of Lars Andersson and Stefano Petti, communicating the passing away of Jens Ove Andreasen and Ulf Glendor^a^
14 September 2021	CSAC voting Summary Yes = 10 No = 0 Undecided = 0
29 October 2021	Proposal accepted by the CSAC
4 March 2022	Proposal Implemented with Modifications by the WHO Team 6
8 March 2022	Final comments provided by the CSAC

a Excerpts available in Appendix S2.

The proposal was finally accepted by the CSAC on October 29, 2021, and was implemented by the WHO on March 8, 2022, with the modifications shown in Table 1. It was retrospectively included in the February 2022 release of the ICD‐11 (available at: https://icd.who.int/browse11/l‐m/en#/http%3a%2f%2fid.who.int%2ficd%2fentity%2f452386362). TDIs were included in block 22 called “Injury, poisoning or certain other consequences of external causes”; category NA, “Injuries to the head.” A new subcategory was generated, comprehensive of all TDIs, called NA0D, “Injury of teeth or supporting structures.” All TDIs were properly described according to the Andreasen classification. The implemented TDI codes are shown in Table 1.

## 3 ANDREASEN CLASSIFICATION INTEGRALLY INCLUDED IN THE ICD‐11

The different appropriateness of TDI classifications between the ICD‐11 versions released in May 2021 and in February 2022, before and after the approval of Proposal #2130 is evident. TDIs finally had their independent and accurate ICD‐11 classification system. The widely acknowledged Andreasen classification of TDIs has been accepted by the WHO integrally, without exemptions.

Unfortunately, the acceptance of Proposal #2130 arrived too late for Jens Ove Andreasen^14^ and Ulf Glendor,^15^ both of whom passed away in 2020. They devoted their lives to alleviate the global burden of TDIs, and although they did not have the opportunity to celebrate this success, the approval of Proposal #2130 is their last gift to the clinical and scientific TDI community.

Hopefully, the inclusion of the Andreasen classification within the ICD‐11 system will help improve the awareness of healthcare providers, policy makers, scientists, and epidemiologists toward TDIs, that could be a little bit less neglected in the future.

## CONFLICT OF INTEREST

The surviving authors confirm that they have no conflict of interest.

## AUTHOR CONTRIBUTIONS

SP, JOA, UG, LA designed and drafted the manuscript. SP and LA approved the submitted version and are accountable for all aspects of the work in ensuring that questions related to the accuracy or integrity of any part of the work are appropriately investigated and resolved. As JOA and UG passed away during the study, they could not approve the submitted version and are no longer accountable for all aspects of the work in ensuring that questions related to the accuracy or integrity of any part of the work are appropriately investigated and resolved.

## Supporting information


Appendix S1

Appendix S2

## Data Availability

The data reported in Tables are accessible on the ICD‐11 Maintenance Platform (available at, https://icd.who.int/dev11/proposals/f/icd/en/ProposalList) typing the name of proposal originator (Petti) to the “Text Search” box.
