# Supplementary material for: NA0D – The new Traumatic Dental Injury classification of the World Health Organization
Source: Dent Traumatol. 2022 Apr 28;38(3):170–4. doi: 10.1111/edt.12753 (PMC10234444; doi:10.1111/edt.12753)
Supplement: Supplementary file 1 — Appendix S1 [file EDT-38-170-s001.docx]

Supplementary Material S1. Proposal #2130 full text

Accessible on the ICD-11 Maintenance Platform (available at, <https://icd.who.int/dev11/proposals/f/icd/en/ProposalList>) typing the name of proposal originator (Petti) to the “Text Search” box

*Rationale*

Traumatic Dental Injuries account for 5% and 17% of all injuries for which people seek treatment in Emergency Services among adults and children, respectively. Their treatment is expensive (2,000,000-5,000,000 USD per million inhabitants), and is more time-consuming than for bodily injuries (Petersson, Andersson & Sörensen, 1997).

According to a meta-analysis, performed by the present working group in 2018 and validated through the Global Burden of Disease (GBD) Study methodology, there are 900,000,000–1,250,000,000 people who live with Traumatic Dental Injuries (Petti, Glendor & Andersson, 2018). Traumatic Dental Injuries are, thus, the fifth most prevalent disease or injury in the world after "permanent caries", "tension-type headache", "iron-deficiency anaemia", and "age-related and other hearing loss", preceding "migraine" and "genital herpes". Paradoxically, Traumatic Dental Injuries are excluded from the list of the 310 most prevalent diseases and injuries considered by the GBD Study (GBD 2015 Disease and Injury Incidence and Prevalence Collaborators, 2016).

The GBD Study is not the only one to overlook Traumatic Dental Injuries. This condition also is neglected by the most important Public Health organizations, such as the US Centers for Disease Control and Prevention (CDC) and even the WHO. Indeed, dental caries, gum diseases, and oral cancer are the only oral diseases taken into consideration by the oral health sections of the CDC and the WHO and no information regarding Traumatic Dental Injuries is reported by the US National Health and Nutrition Examination Survey, the National Health Interview Survey, or by the Country Oral Health Profiles of the WHO (Petti, Andreasen, Glendor & Andersson, 2018).

Such a lack of awareness toward Traumatic Dental Injuries is pervasive in general (i.e., non-dental) Emergency Services. Indeed, prompt and proper treatment of injured teeth is often not carried out due to inadequate knowledge of assessment and treatment protocols among non-dental healthcare workers. Inadequate assessment and delayed appropriate treatment frequently lead to complications, such as pulpitis, pulp necrosis, root resorption, and tooth extraction. Poor treatment protocols have unsatisfactory outcomes for both patients and practitioners (Moule & Cohenca, 2016). For example, in Serbian Emergency Services, Traumatic Dental Injury treatment delay occurred in 90% of events during after-hours and only in 39% of events during office-hours, while the median times from injury to treatment were 48 and 2 hours during after-hours and office-hours, respectively, because dental healthcare providers were available only during office-hours (Vukovic et al, 2016). In addition, only 4% of physicians working in Emergency room facilities in Israeli hospitals are able to appropriately treat injured teeth (Holan & Shmueli, 2003). Correct diagnosis in Emergency Services leads to appropriate treatment of injured teeth, as shown in the Dental Trauma Guide (available at, https://dentaltraumaguide.org/).

However, correct diagnosis of Traumatic Dental Injuries using the ICD system is challenging for untrained healthcare workers, as shown in an Emergency Department at the Great Western Hospital of Swindon, UK, among patients with facial trauma. Indeed, the UK National Health Service (NHS) uses the International Classification of Diseases (ICD) for diagnostic coding (at the time of the survey, in 2014, the ICD-10 was in use) and regrettably, only 25% of the visits were coded with sufficient detail. Specifically, 20% of patients were coded with the least specific code S09.9 "Unspecified injury of head", other patients were coded with T14.9 "Injury, unspecified". These codes give no information as to the cause, the nature and the needed treatment of the injury. A further 27% of patients were coded with either S01.8 "Open wound of other part of head", T14.1 "Open wound of unspecified body region", S00.8 "Superficial injury of other parts of head", or S01.9 "Open wound of head, part unspecified". These codes classify the nature of the wound but again give no information as to the cause of the injury, thus leading to inappropriate treatments or lack of treatment. There were also 7% patients who had no code, while 20% received "System generated" codes, which means that no code at all was given (Lello, Allen & Haig 2015).

The freshly released ICD-11 did not fix these important diagnostic problems regarding Traumatic Dental Injuries. Indeed, some types of injuries, namely, Infraction, Subluxation and Concussion, have not an appropriate code, thus leaving NA0Z “Injuries to the head, unspecified" as the only permitted code. As previously reported, this lack of classification is responsible for problems to healthcare workers in the Emergency Services and to injured patients, as the latter could not receive adequate diagnosis (or receive no diagnosis at all) and could develop complications due to missing or improper dental therapy. In many countries, patients without diagnosis cannot apply for refund from insurance companies or get access to public dental healthcare, while healthcare workers and patients could may face medico-legal problems. These drawbacks may even lead to inequalities in Traumatic Dental Injury treatment and prevention (Petti et al, Lancet Glob Health 2018). For example, high social vulnerability –a marker of socio-economic status based on factors such as, access to work, income, education, legal assistance, etc.- doubles the probability that Brazilian children with Traumatic Dental Injuries do not receive appropriate dental healthcare or any care at all (Bendo, Vale, Figueiredo, Pordeus & Paiva 2012).

The inadequate classification of Traumatic Dental Injuries according to ICD is also evident from the analysis of the world scientific literature. A comprehensive literature search, performed on December 7th 2018, with the keywords [“traumatic dental injury” AND “ICD”] in “All fields”, and without time restriction, using the most important databases, provided the following results,

• Web of Science, 0 documents (using “traumatic dental injury” alone 180 documents, using “ICD” alone 30,970 documents);

• PubMed, 0 documents (using “traumatic dental injury” alone 144 documents, using “ICD” alone 32,175 documents);

• Scopus, 51 documents –in these documents, however, ICD did not refer to Traumatic Dental Injury diagnosis but to other diseases and conditions possibly associated to Traumatic Dental Injuries (using “traumatic dental injury” alone 3,003 documents, using “ICD” alone 138,618 documents).

A similar search focusing on “oral cancer” provided 40 documents (Web of Science), 39 documents (PubMed), and 410 documents (Scopus). This literature search shows that ICD is poorly used, or not used at all, by the indexed scientific literature regarding Traumatic Dental Injuries.

Cumulatively, these data demonstrate that improper ICD classification of Traumatic Dental Injuries may safely explain why one of the most frequent diseases in the world, is ignored by the main Public Health organizations and the scientific literature, and is a neglected condition in non-dental healthcare settings. Even the ICD-DA, that is, the Application of the ICD to Dentistry and Stomatology (the last edition, released in 1994, is available at http://apps.who.int/iris/bitstream/handle/10665/40919/9241544678_eng.pdf;jsessionid=04126F7A073CFE1B2274183B83240CD2?sequence=1) cannot help overcome these problems. Indeed, once again there are no codes at all for the following injuries,

• Infraction: incomplete fracture of the enamel without loss of tooth structure;

• Concussion: injury without abnormal loosening or displacement of the tooth but the tooth is tender to touch;

• Subluxation: injury with abnormal loosening but without displacement of the tooth.

Indeed, the ICD-DA code S02.5 ("Fracture of tooth” –page 114) implies the loss of tooth structure and cannot be used for Infraction, while the code S03.2 (“Dislocation of tooth” –page 115) implies tooth displacement and cannot be used for Concussion and Subluxation.

The current proposal was made by an international working group of experts in Dental Traumatology. Namely, Prof. Stefano Petti (who formally submitted this proposal to the scientific board of the “International Classification of Diseases” on behalf of the other colleagues), oral epidemiologist, Deputy Editor of the scientific journal "Oral Diseases"; Prof. Jens Ove Andreasen, author, in 1970, of the Dental Trauma classification which is currently acknowledged worldwide both in dental healthcare settings and in scientific literature (Andreasen, 1970), this classification is accepted by the International Association of Dental Traumatology (IADT) and is the basis of the Dental Trauma Guide, the international evidence-based treatment guide for Traumatic Dental Injuries; Prof. Ulf Glendor, past Editor of the scientific journal "Dental Traumatology", board member of IADT and co-author of the Dental Trauma Guide; Prof. Lars Andersson, past Editor-in-Chief of the scientific journal "Dental Traumatology", past President of IADT, and co-author of the Dental Trauma Guide. This group also authored chapter 11 of the “Textbook and Color Atlas of Traumatic Injuries to the Teeth, 5th Edition” (Andersson, Petti, Day, Kenny, Glendor & Andreasen, 2019) that reports the Andreasen classification of Traumatic Dental Injuries.

*Problems with the current ICD-11*

Problem #1

Within the code NA02.6 (Fracture of tooth), dental fractures lack description; the listed subtypes do not apply to teeth; there is no applicable code for Infraction

ICD-11 CLASSIFICATION FOR DENTAL FRACTURES

22-Injury; Injuries to the head; NA02 Fractures of skull and facial bones; NA02.6 Fracture of tooth (former ICD-10 and ICD-DA: S02.5).

Description: “this entity does not have a definition at the moment.

Inclusions: Broken tooth.

Exclusions: Nontraumatic fracture of tooth.

All Index Terms: • Fracture of tooth • Broken tooth • enamel and dentine fracture • chipped tooth • Enamel fracture only • horizontal traumatic fracture of tooth • Fracture of crown of tooth, enamel and • dentin, with pulp exposure • Fracture of crown of tooth, enamel only • Fracture of tooth root • Fractured tooth with pulp involvement.

Fracture subtype (use additional code, if desired): XJ36W Avulsion fracture; XJ2EL Bucket handle or corner fracture; XJ76E Buckle fracture; XJ7ZH Burst fracture; XJ1Z6 Comminuted fracture; XJ1PP Compound fracture; XJ778 Compression fracture; XJ9UB Depressed fracture; XJ69V Dislocated fracture; XJ8PQ Displaced fracture; XJ0QE Elevated fracture; XJ5N9 Fissured fracture; XJ45W Greenstick fracture; XJ7AT Impacted fracture; XJ4PE Infected fracture; XJ392 Linear fracture; XJ6RL Longitudinal fracture; XJ4CX Missile fracture; XJ4FU Osteochondral fracture; XJ3HH Physeal fracture; XJ64N Puncture fracture; XJ909 Simple fracture; XJ9XQ Slipped epiphysis fracture; XJ967 Spiral fracture; XJ5V7 Transverse fracture; XJ6NA Wedge fracture; XJ8QL Fracture with foreign body.

RELATED PROBLEMS

• There is no Description of code NA02.6 “Fracture of tooth”.

• None of the listed fracture subtypes apply to fractured teeth.

• Dental fracture subtypes are listed as Index Terms.

• There is no applicable code or Index Term for Infraction (incomplete fracture of tooth without substance loss).

Problem #2

Tooth dislocation (NA03.2) and dental contusion (NA00.7) are classified using two different codes, as if they were two different entities but dislocation is a type of contusion; description is wrong; there are no applicable codes for Concussion and Subluxation

ICD-11 CLASSIFICATION FOR TOOTH DISLOCATION

22-Injury; Injuries to the head; NA03 Dislocation or strain or sprain of joints or ligaments of head; NA03.2 Dislocation of tooth (former ICD-10 and ICD-DA: S03.2).

Description: “#DRAFT# This is a violent shift of a tooth with damage of a periodontium. Partial or complete detachment of a tooth from its socket”.

All Index Terms: • Dislocation of tooth • luxation of tooth • avulsion of tooth.

Postcoordination: Add detail to “Dislocation of tooth”, Specific anatomy (use additional code, if desired); from this section it is possible to digit the word “teeth” and the code XA4GG3 for unspecified permanent teeth can be used, other codes for specific teeth (not all teeth) and primary teeth are available.

TOOTH DISLOCATION RELATED PROBLEMS

• There are no dislocation subtypes.

• Dislocation subtypes are listed as Index Terms, but the list is incomplete.

• There are no applicable codes or Index Term for Concussion and Subluxation (dental contusions with damage of the periodontium but without tooth dislocation).

ICD-11 CLASSIFICATION FOR DENTAL CONTUSION

22-Injury; Injuries to the head; NA00 Superficial injury of head; NA00.7 “Contusion of other or unspecified sites of head” (former ICD-10 and ICD-DA: S00.59 “Superficial injury of lip and oral cavity, unspecified”).

Description: “This entity does not have a definition at the moment. You may suggest a definition using our Proposal System available under the Contributions menu”.

All Index Terms: • Contusion of other or unspecified sites of head • head contusion • Contusion of supraorbital region • Contusion of forehead.

Postcoordination: Add detail to “Contusion of other or unspecified sites of head”, Specific anatomy (use additional code, if desired); from this section it is possible to digit the word “teeth” and the code XA6CZ2 for unspecified teeth can be used, other codes for permanent and primary teeth are available.

DENTAL CONTUSION RELATED PROBLEMS

• There is no Description of code NA00.7 “Contusion of other or unspecified sites of head”.

• There is no reference to teeth.

• Concussion and Subluxation (dental contusions with no concurrent tooth dislocation and, therefore, cannot be coded with NA03.2) must necessarily be coded with NA00.7, but there are no appropriate subtypes, Index Terms and definitions.

OVERALL PROBLEM

• Dental contusions and tooth dislocations actually are two forms of the same type of Traumatic Dental Injury known as “Contusion” or “Injury to the periodontium”. The entity of the impact on the tooth may result in tooth dislocation and even dental avulsion, but the periodontium can also be damaged without dislocation, and these are the cases of Concussion and Subluxation. It is necessary to unify these types of contusions into one specific code.

Problem #3

Dental Contusions and Dental Fractures are in different blocks

PROBLEM

As shown with Problems #1 ad #2, dental fractures are in the NA02 block, while dental contusions are in the NA03 and NA00 blocks.

Concluding Remarks

These problems, already present in the past ICD versions and in the ICD-DA, often result in misclassifying Traumatic Dental Injuries using nonspecific codes, such as NA0A.Y “Other specified injuries of head”, or NA0Z “Injuries to the head, unspecified” (20% of facial injuries are classified by healthcare workers in Emergency Services in UK using the former ICD-10 code S09.9 “Unspecified injury of head” (Lello, Allen & Haig, 2015) that in the current version would be NA0Z), or not classifying them at all (20% of facial injuries are not classified at all by healthcare workers in Emergency Services in UK). These problems could be solved generating a unique, specific and detailed block for Traumatic Dental Injuries.

This block would give the opportunity to healthcare workers who are not expert in dentistry to perform adequate assessments of Traumatic Dental Injuries with consequent benefits to patients, healthcare workers, and the healthcare systems, it would help increase the awareness toward this frequent and neglected condition, which could be included in the list of diseases by Public Health organizations, such as WHO and CDC and by the GBD Study.

Stefano Petti, Sapienza University of Rome (Italy)

Jens Ove Andreasen, Copenhagen University Hospital, Copenhagen (Denmark)

Ulf Glendor, Malmö University, Malmö (Sweden)

Lars Andersson, Malmö University, Malmö (Sweden)

*References*

• Andersson L, Petti S, Day P, Kenny K, Glendor U, Andreasen JO. Classification, Epidemiology and Etiology. In: Andreasen JO, Andreasen FM, Andersson L Eds. Textbook and Color Atlas of Traumatic Injuries to the Teeth, 5th Edition. John Wiley & Sons Ltd, 2019; pp. 252-294.

• Andreasen JO. Etiology and pathogenesis of traumatic dental injuries. A clinical study of 1,298 cases. Scand J Dent Res 1970;78(4):329-342.

• Bendo CB, Vale MP, Figueiredo LD, Pordeus IA, Paiva SM. Social vulnerability and traumatic dental injury among Brazilian schoolchildren: a population-based study. Int J Environ Res Public Health 2012;9(12):4278-4291.

• GBD 2015 Disease and Injury Incidence and Prevalence Collaborators. Global, regional, and national incidence, prevalence, and years lived with disability for 310 diseases and injuries, 1990-2015: a systematic analysis for the Global Burden of Disease Study 2015. Lancet 2016;388(10053):1545-1602.

• Holan G, Shmueli Y. Knowledge of physicians in hospital emergency rooms in Israel on their role in cases of avulsion of permanent incisors. Int J Paediatr Dent 2003;13(1):13-19. doi:10.1046/j.1365-263x.2003.00414.x.

• Lello S, Allen P, Haig S. Aetiology of paediatric facial trauma at a UK District General Hospital. Oral Surg 2015;8(4):208-216. doi:10.1111/ORS.12143.

• Moule A, Cohenca N. Emergency assessment and treatment planning for traumatic dental injuries. Aust Dent J 2016;61 Suppl 1:21-38. doi:10.1111/adj.12396.

• Petersson EE, Andersson L, Sörensen S. Traumatic oral vs non-oral injuries. Swed Dent J 1997;21(1-2):55-68.

• Petti S, Glendor U, Andersson L. World traumatic dental injury prevalence and incidence, a meta-analysis-One billion living people have had traumatic dental injuries. Dent Traumatol 2018;34(2):71-86. doi:10.1111/edt.12389.

• Petti S, Andreasen JO, Glendor U, Andersson L. The fifth most prevalent disease is being neglected by public health organisations. Lancet Glob Health 2018;6(10):e1070-e1071. doi:10.1016/S2214-109X(18)30380-2.

• Vukovic A, Vukovic R, Markovic D, Soldatovic I, Mandinic Z, Beloica M, Stojan G. After-Hours Versus Office-Hours Dental Injuries in Children: Does Timing Influence Outcome? Clin Pediatr 2016;55(1):29-35. doi:10.1177/0009922815584214.

*Detailed Explanation of the Proposal*

**• Deleting the code NA02.6 “Fracture of tooth” (former ICD-10 and ICD-DA: S02.5)**

**• Deleting the code NA03.2 “Dislocation of tooth” (former ICD-10 and ICD-DA: S03.2)**

**• Generating a new Block, NA10, possibly called “TRAUMATIC DENTAL INJURY” OR “INJURY TO** **TEETH”**

Proposed position: After NA09 “Traumatic amputation of part of head” and before NA0A “Certain specified injuries of head”

Detailed description is following:

NA10 “Traumatic dental injury” OR “Injury to teeth”

Ancestors: 22 Injury, poisoning or certain other consequences of external causes; Injuries to the head; NA10 “Traumatic dental injury” OR “Injury to teeth”.

Parent: Abfraction DA08.13

Description: Damage inflicted on the surface of the tooth as the direct or indirect result of an external force, with disruption of continuity of the tooth substance, and/or impairment of the tooth-supporting structures (periodontium)

Inclusions

• Cracked tooth

• Enamel chipping

• Broken tooth

• Loose tooth

• Displaced tooth

• Tooth exarticulation

Exclusions

• Dental caries DA08

• Excessive attrition of teeth DA08.10

• Abrasion of teeth DA08.11

• Erosion of teeth DA08.12

• Chronic dental injuries DA08.2

• Nontraumatic fracture of tooth DA08.3

• Certain specified disorders of teeth or supporting structures DA0A

All Index Terms: None

NA10.1 “Injuries to the hard dental tissues and the pulp”

Ancestors: 22 Injury, poisoning or certain other consequences of external causes; Injuries to the head; NA10 “Traumatic dental injury” OR “Injury to teeth”; NA10.1 “Injuries to the hard dental tissues and the pulp”

Description: Damage inflicted on the surface of the tooth as the direct or indirect result of an external force, with disruption of continuity of the tooth substance

Postcoordination: Add detail to Injuries to the hard dental tissues and the pulp

Type of injury (use specific codes if desired):

• NA10.10 Enamel infraction → An incomplete fracture (crack) of the enamel without loss of tooth substance

• NA10.11 Enamel fracture → Uncomplicated crown fracture. A fracture with loss of tooth substance confined to the enamel

• NA10.12 Enamel-dentin fracture → Uncomplicated crown fracture. A fracture with loss of tooth substance confined to enamel and dentin, but not involving the pulp

• NA10.13 Complicated crown fracture → A fracture involving enamel and dentin, and exposing the pulp

• NA10.14 Uncomplicated crown-root fracture → A fracture involving enamel, dentin and cementum, but not exposing the pulp

• NA10.15 Complicated crown-root fracture → A fracture involving enamel, dentin and cementum, and exposing the pulp

• NA10.16 Root fracture → A fracture involving dentin, cementum and the pulp. Root fractures can be further classified according to displacement of the coronal fragment

Specific anatomy:

XA6CZ2 Teeth

NA10.2 “Injuries to the periodontal tissues”

Ancestors: 22 Injury, poisoning or certain other consequences of external causes; Injuries to the head; NA10 “Traumatic dental injury” OR “Injury to teeth”; NA10.2 “Injuries to the periodontal tissues”

Description: Damage inflicted on the surface of the tooth as the direct or indirect result of an external force, with impairment of the tooth-supporting structures (periodontium)

Postcoordination: Add detail to Injuries to the Injuries to the periodontal tissues

Type of injury (use specific codes if desired):

• NA10.20 Concussion → An injury to the tooth-supporting structures without abnormal loosening or displacement of the tooth, but with marked reaction to percussion

• NA10.21 Subluxation → An injury to the tooth-supporting structures with abnormal loosening, but without displacement of the tooth

• NA10.22 Extrusive luxation → Peripheral dislocation, partial avulsion. Partial displacement of the tooth out of its socket

• NA10.23 Lateral luxation → Displacement of the tooth in a direction other than axially. This is accompanied by comminution or fracture of the alveolar socket

• NA10.24 Intrusive luxation → Central dislocation. Displacement of the tooth into the alveolar bone. This injury is accompanied by comminution or fracture of the alveolar socket

• NA10.25 Avulsion → Exarticulation. Complete displacement of the tooth out of its socket

Specific anatomy:

XA6CZ2 Teeth
