# Supplementary material for: NA0D – The new Traumatic Dental Injury classification of the World Health Organization
Source: Dent Traumatol. 2022 Apr 28;38(3):170–4. doi: 10.1111/edt.12753 (PMC10234444; doi:10.1111/edt.12753)
Supplement: Supplementary file 2 — Appendix S2 [file EDT-38-170-s002.docx]

Supplementary Material S2. Excerpts from the Comments to the Proposal #2130

Full texts accessible on the ICD-11 Maintenance Platform (available at, <https://icd.who.int/dev11/proposals/f/icd/en/ProposalList>) typing the name of proposal originator (Petti) to the “Text Search” box

*QED-WHO-CC (3 March, 2021)*

Dr. Benoit Varenne, Dental Officer

Noncommunicable Diseases Department

World Health Organization

Geneva

Dear Dr. Varenne,

We have reviewed the technical proposal and scientific support regarding the inclusion of dental injuries in the International Classification of Diseases (ICD-11) reporting system. The proposal includes a good summary of the scientific evidence supporting the development of independent coding with the following public health benefits:

1) Appropriate diagnostic codes to describe and analyze socio-demographic and environmental factors associated with dental and soft-tissue injuries.

2) The burden of dental and soft-tissue injuries in populations.

3) The preventive effects of interventions.

We agree with the proposal that dental and soft-tissue injuries have dedicated codes because these events may have more than one clinical presentation. Also, we support that the proposed coding expands those used in clinical settings already familiar to many clinicians.

In conclusion, we found that the proposal is sound and self-standing. We would like to add some minimal suggestions:

1) There have been some epidemiological studies on dental injuries in some parts of the world. These are old publications (in the 1980s, 1990s, and even 2000s) using similar coding (Andreassen’s classification) as those proposed, and Bhat’s code for dental injuries in the US used in NHANES. We think that this may support the proposed coding.

2) It could be convenient to indicate that dental injuries may fall under both classifications of intentional and non-intentional injuries and highlight that the entire population is at risk.

Please, let us know if you or the authors require additional clarification,

Sincerely,

Eugenio D. Beltran

Habib Benzian

Richard Niederman

Eugenio Beltran 2021-Mar-03 - 20:15 UTC

*Benoit Varenne (4 March, 2021)*

I agree with the review and recommendations made by the WHO CC NYU Team related to the inclusion of dental injuries into ICD-11. Bet regards. Benoit Varenne

benoit varenne 2021-Mar-04 - 17:22 UTC

*CSAC Small Group (5 May, 2021)*

The CSAC Small Group has reviewed this proposal and recommends it be accepted:

• Delete NA02.6 Fracture of tooth and move all index terms

• Delete NA03.2 Dislocation of tooth and move all index terms

• Create new grouping for ‘Injury to teeth’ with the primary parent of ‘Injuries to the head’, subclassified by two new codes ‘Injuries to hard dental tissues and pulp’ and ‘Injuries to the periodontal tissues’.

• Add postcoordination for the teeth as specific anatomy to both new codes.

CSAC Secretariat 1 2021-May-05 - 13:58 UTC

*Lars Andersson and Stefano Petti (9 August, 2021)*

Unfortunately, Jens Andreasen, one of the coauthors of this proposal, and the creator of the classification system of Traumatic Dental Injuries, passed away a few months ago (you can read his obituary written by Lars Andersson, another coauthor of the present proposal, for the journal Dental Traumatology here, https://onlinelibrary.wiley.com/doi/epdf/10.1111/edt.12641 -by the way, two other scientific dental journals, namely, Journal of Endodontics and Oral Diseases, also republished this obituary, since Jens really is universally acknowledged as the "Father" of Dental Traumatology).

Jens spent more than a decade in trying to modify the current incomplete ICD system part regarding dental traumatology, but he failed, because he was unaware of the maintenance platform. Thus, the release of the ICD-11 system was a unique opportunity to provide a clear, and comprehensive classification of traumatic dental injuries, overcoming the problems of the past classification, where lesions to soft tissues and to hard tissues were located in two different blocks, and where some types of dental injury were not included.

We submitted this proposal in January 2020, almost three years ago, and we are happy to see how the process is going on. We truly hope that it will come through, it would be the last gift of Jens to the scientific community.

Unfortunately, this comment is signed by only two of the four original authors of this proposal, since Ulf Glendor, also passed away a few months before Jens at the age of 71 (see the obituary here, https://onlinelibrary.wiley.com/doi/epdf/10.1111/edt.12565, written at that time by Jens himself and Lars).

Stefano Petti and Lars Andersson

Stefano Petti 2021-Aug-09 - 09:43 UTC

*CSAC Final Comments (8 March, 2022)*

The Classifications and Statistics Advisory Committee reviewed this proposal and accepted it with modification. THe proposal has been implemented with the following additional modifications:

* For the entity NA0D Injury of teeth', a modification of title has been suggested to 'Injury of teeth or supporting structures'.

* Add index term 'Dental contusion' to NA0D.10 'Concussion of periodontal tissue'.

CSAC Secretariat 1 2022-Mar-08 - 05:28 UTC
